# What binds us? Inter-brain neural synchronization and its implications for theories of human consciousness

**DOI:** 10.1093/nc/niaa010

**Published:** 2020-06-11

**Authors:** Ana Lucía Valencia, Tom Froese

**Affiliations:** n1 Psychobiology and Neuroscience Department, Faculty of Psychology, National Autonomous University of Mexico, Mexico City, Mexico; n2 Embodied Cognitive Science Unit, Okinawa Institute of Science and Technology Graduate University, Okinawa, Japan

**Keywords:** neural synchronization, hyperscanning, electroencephalography, EEG, social interaction, human mind, consciousness

## Abstract

The association between neural oscillations and functional integration is widely recognized in the study of human cognition. Large-scale synchronization of neural activity has also been proposed as the neural basis of consciousness. Intriguingly, a growing number of studies in social cognitive neuroscience reveal that phase synchronization similarly appears across brains during meaningful social interaction. Moreover, this inter-brain synchronization has been associated with subjective reports of social connectedness, engagement, and cooperativeness, as well as experiences of social cohesion and ‘self-other merging’. These findings challenge the standard view of human consciousness as essentially first-person singular and private. We therefore revisit the recent controversy over the possibility of extended consciousness and argue that evidence of inter-brain synchronization in the fastest frequency bands overcomes the hitherto most convincing sceptical position. If this proposal is on the right track, our understanding of human consciousness would be profoundly transformed, and we propose a method to test this proposal experimentally.

## Introduction

In social cognitive neuroscience, a shift toward embodied, enactive, and participatory approaches has started to take place, moving away from individual brains and focusing on a person’s interaction with the environment. In the context of social cognition a ‘second-person’ approach (Hari and Kujala 2009; Dumas 2011; Hasson *et al.* 2012; Hari *et al.* 2013; Schilbach *et al.* 2013; Redcay and Schilbach 2019) has gained popularity, emphasizing the interactive nature of human cognition (Szymanski *et al.* 2017; Varela *et al.* 2017), and even challenging the individualist notion of human experience (Thompson 2001; Hari and Kujala 2009; Torrance 2009; Dumas 2011; Kirchhoff 2014; Froese 2018; Kirchhoff and Kiverstein 2019).

These approaches open the space for neuroscience to experimentally address the constitutive role of brain-to-brain relationships in shaping the mind during moment-to-moment interactions (Hari and Kujala 2009; Dumas 2011; Hasson *et al.* 2012; Redcay and Schilbach 2019). Important philosophical topics, such as collective intentionality (Searle *et al.* 1990), can therefore be revisited.

However, there is also some notable resistance towards moving into this uncharted territory. Even the principal architect of the hypothesis of the extended mind, Andy Clark, has remained sceptical about whether this hypothesis can be generalized to ‘the extended conscious mind’ (Clark 2009). For Clark, the most promising type of argument appeals to dynamic entanglement plus a unique temporal signature, which is based on a popular class of theories of conscious experience that require very fine-grained processes of temporal coordination to bind together meaningful neural activity. Yet he noted the lack of evidence for such processes operating outside an individual’s nervous system, and hence concluded that the case for the extended conscious mind ‘is at best unproven’ (Clark 2009). A decade on from Clark’s influential assessment it has become clear that the current best evidence from social cognitive neuroscience entails a more optimistic conclusion, with potentially far-reaching implications about what it means to be human.

Behavioural studies in psychology have consistently shown that synchrony during joint action (such as rocking, marching, walking, or dancing) promotes cooperative ability and increases empathy, liking, rapport, and prosocial behaviour (Hari and Kujala 2009; Hove and Risen 2009; Wiltermuth and Heath 2009; Valdesolo *et al.* 2010; Valdesolo and DeSteno 2011; Mogan *et al.* 2017). From such studies, it has been suggested that dynamics of neuronal coupling could play an important role in the emergence of such interactive synchrony (Wilson and Wilson 2005; Dumas *et al.* 2011; Hasson *et al.* 2012). Importantly, the development of the hyperscanning technique by Montague *et al.* (2002) has allowed for the measurement and analysis of such inter-brain dynamics (Babiloni and Astolfi 2014; Czeszumski *et al.* 2020). Using functional magnetic resonance imaging (fMRI), functional near-infrared spectroscopy (fNIRS), electroencephalography (EEG) or magnetoencephalography (MEG), hyperscanning paradigms simultaneously record the brain activity of two or more individuals, thus permitting the assessment of neural activity during real-time social interaction (Babiloni and Astolfi 2014; Czeszumski *et al.* 2020).

In the past, the brain of only one participant in a group or dyad would be recorded at a time, obscuring any emerging phenomena of reciprocal embodied interaction (Dumas 2011; Hasson *et al.* 2012; Czeszumski *et al.* 2020). But as Montague very simply phrased it, ‘studying social interactions by scanning the brain of just one person is analogous to studying synapses while observing either the presynaptic neuron or the postsynaptic neuron, but never both simultaneously’ (Montague *et al.* 2002). Hyperscanning allows for the investigation of neural relations at the intra- and inter-brain level (Schilbach *et al.* 2013; Czeszumski *et al.* 2020), making it a relevant technique for understanding the neural basis of social interaction.

Keeping in mind that intra-brain neural oscillations are known to play a critical role in cognitive processes (since they represent the precise timing of neural activity Buzsáki 2006; Sauseng and Klimesch 2008) and, more importantly, that large-scale phase synchronization has further been proposed as the neural basis of consciousness (Crick and Koch 1990; Thompson and Varela 2001; Ward 2003; Engel *et al.* 2016) (see below), reports of modifications on the ongoing oscillatory activity of several individuals due to social interaction (as measured using EEG-based hyperscanning) have important implications for our study of human behaviour.

This opinion piece aims to examine findings of EEG-based hyperscanning studies and highlight the importance of inter-brain neural synchronization for the study of consciousness. We discuss findings of brain-to-brain synchronization during cooperative social interaction, revealing that this phenomenon is not a general effect of a shared environment, but an emergent property of specifically social dynamics. This, together with the subjective reports of social connectedness and engagement that often accompany said findings, leads to a new outlook regarding the importance of inter-brain neural synchronization in understanding the nature of human consciousness.

## Oscillations, synchronization and consciousness

Neural oscillations are considered to be causally responsible for information transfer and integration (Rosenblum *et al.* 2001; Herrmann *et al.* 2016) since they can change the functional interactions between areas of the brain (Varela *et al.* 2001; Buzsáki 2006; Sauseng and Klimesch 2008; Fries 2015; Bonnefond *et al.* 2017), most probably through phase synchronization (Varela *et al.* 2001; Ward 2003; Buzsáki 2006; Uhlhaas *et al.* 2009). Since the phase of an oscillation reflects the exact timing of neural activity (Singer 1993; Buzsáki 2006; Cohen 2014), phase synchronization—both within and across EEG frequency bands—represents a window of functional communication and integration between neuronal populations (Sauseng and Klimesch 2008).

It is established that cognitive processes require the transient coalition of several, widely distributed, and interacting neuronal groups (Thompson and Varela 2001; Ward 2003; Siegel *et al.* 2012) and that this large-scale dynamical integration is accomplished precisely by phase synchronization of cell assemblies (Hebb 1949; Nicolelis *et al.* 1997; Rodriguez *et al.* 1999; Varela *et al.* 2001; Kelso and Engstrom 2006; Sauseng and Klimesch 2008; Kelso 2009; Tognoli and Kelso 2009; Fries 2015; Engel *et al.* 2016) (of course, synchronization is not the only mechanism underlying effective cognitive processing; for a discussion on importance of phase *de*synchronization, see Tognoli and Kelso 2009 and Varela *et al.* 2001). Analogously, the neural substrates of consciousness may not be localized to a single region or network of a person’s brain (Kelso 1995; Thompson and Varela 2001; Engel *et al.* 2016). Two decades ago, Crick and Koch (1990) suggested a link between synchronized neural oscillations and consciousness; today several authors support the claim that the neural basis of consciousness (specifically of phenomenal consciousness, i.e., the integrated flow of experience Block 1995) is likely to be at the level of large-scale interactions over several frequency bands of oscillatory neural activity (Engel *et al.* 1999, 2016; Buzsáki 2006; Thompson and Varela 2001; Varela *et al.* 2001; Ward 2003; Melloni *et al.* 2007; Uhlhaas *et al.* 2009; Revonsuo 2014). For example, Llinás suggested that our subjectivity is generated by temporally coherent neural activity (‘It binds, therefore I am’ Llinás 2001), while Engel and Singer proposed that neural synchronization could be the mechanism of different aspects of consciousness, and even what generates the global unity of the self and the world (Engel *et al.* 1999; Revonsuo 2014).

A large number of evidence further suggest that changes in the patterns of neuronal coherence, both locally and across regions, may lead to changes in mental functioning and the contents of consciousness (Engel *et al.* 2016). Abnormal oscillatory synchronization has not only been associated with conditions such as epilepsy, Parkinson’s disease, Huntington’s disease, essential and cerebellar tremors and coma (Schnitzler and Gross 2005; Buzsáki 2006)—it has also been linked to psychiatric disorders like schizophrenia. Both hallucinatory symptoms and the fragmented nature of these patients’ experience have been related to a disruption of synchronization (Tononi and Edelman 2000; Lisman and Buzsaki 2008; Uhlhaas *et al.* 2009; Uhlhaas and Singer 2010; Mellin *et al.* 2018; Ahn *et al.* 2019).

As a process crucial for consciousness, oscillations can cut across brain–body–world divisions, rather than being limited to neural activity in the head (Thompson and Varela 2001). An interesting and well-known example of their role in guiding cognition and conscious experience in the world is the adjustment of ongoing neural rhythms to external stimuli, also known as phase locking (Sauseng and Klimesch 2008). Critical to synchronization by oscillation (Buzsáki 2006), phase locking to external stimuli has been described as a ‘gating’ mechanism that can amplify or reduce neuronal responses to the events of an information stream (Bonnefond and Jensen 2012). It has also been related to task performance and conscious perception (Lakatos *et al.* 2008; Bonnefond and Jensen 2012; Ronconi *et al.* 2017; Solís-Vivanco *et al.* 2018). Therefore, its results are crucial for a person’s ability to retrieve information about the world and successfully interact with her/his environment (Hasson *et al.* 2012). In contrast, schizophrenia patients display reduced alignment of neural activity to external stimuli (Lakatos *et al.* 2008; Lakatos *et al.* 2013), and it has also been proposed that the disconnection between self and environment in these patients could be due to this abnormality (Lakatos *et al.* 2013).

## Neural synchronization beyond an individual brain

The continuous modification of internal oscillatory dynamics is not limited to an individual’s interaction with the physical environment. A growing body of research examining neuronal processes in interacting individuals has revealed that social dynamics also play an important role in neuronal rhythms (Balconi and Vanutelli 2017; Stevens and Galloway 2017; Mu *et al.* 2018). For example, studies have revealed that individuals on the autistic spectrum disorder, who often have substantial problems connecting socially (Hari and Kujala 2009; Marsh *et al.* 2013; Redcay and Schilbach 2019), show a lack of neural synchronization with others (Tanabe *et al.* 2012; Salmi *et al.* 2013).

Furthermore, tasks requiring cooperation, coordination and joint attention in non-clinical individuals demonstrate that greater inter-brain oscillatory synchronization is associated with enhanced performance and can predict team efficiency (Mu *et al.* 2017; Szymanski *et al.* 2017; Balconi and Vanutelli 2018), and that tasks with a greater need for cooperation are associated with a higher level of inter-brain synchronization (Bezerianos *et al.* 2015; Mu *et al.* 2016; Sinha *et al.* 2016; Mu *et al.* 2017; Szymanski *et al.* 2017; Hu *et al.* 2018).

The behavioural gains associated with higher inter-brain synchronization subsequently make hyperscanning, and in particular EEG-based hyperscanning (Hari and Kujala 2009), specifically relevant for evaluating the neural oscillatory dynamics associated with social interactions (Liu *et al.* 2018).

As with other techniques, EEG-based hyperscanning is carried out during a real-time interaction paradigm (Babiloni and Astolfi 2014). Most commonly the paradigms used include tasks such as playing guitars in duets (Lindenberger *et al.* 2009; Sänger *et al.* 2012), imitation tasks (Dumas *et al.* 2010; Yun *et al.* 2012), visual search tasks (Szymanski *et al.* 2017), cooperation–competition games (Astolfi *et al.* 2010; De Vico Fallani *et al.* 2010; Cui *et al.* 2012; Sinha *et al.* 2016; Pan *et al.* 2017; Hu *et al.* 2018), verbal or motor interaction (Pérez *et al.* 2017), amongst others (for reviews see: Dumas *et al.* 2011; Babiloni and Astolfi 2014; Balconi and Vanutelli 2017; Liu *et al.* 2018; Czeszumski *et al.* 2020). Each member of the group or dyad is instrumented with the desired scalp electrode channels from separate EEG devices, and all devices are controlled through an online server (so as to make sure neural recordings run in parallel). A key advantage of EEG-based hyperscanning is that it is capable of maintaining high ecological validity, while capturing changes in the phase relationship between oscillatory signals of individual brains in the millisecond range (Mu *et al.* 2018; Czeszumski *et al.* 2020).

Inter-brain phase synchronization analysis under this technique uses adapted versions of intra-brain estimators, such as the Phase Locking Value (PLV) (Lachaux *et al.* 1999), Inter-brain Phase Coherence (IPC) (Lindenberger *et al.* 2009), and Partial Directed Coherence (PDC) (Astolfi *et al.* 2010). While PLV and IPC are measures of similarity between neural signals, PDC is used to estimate causal links between brains (Czeszumski *et al.* 2020). Other measures used include the Circular Correlation Coefficient (Ccorr), Coherence (COH) measure, Pearson’s Correlation Coefficient, Spearman’s Correlation Coefficient, Total Interdependence (TI, and Wavelet Transform Coherence (WTC). Each estimator has advantages and limitations (Burgess 2013; Czeszumski *et al.* 2020); if used properly, they can reveal the existence of a functional relationship between neural signals of individual brains.

## The functional meaning of inter-brain synchronization

‘True’ synchronization occurs when two oscillators reciprocally adjust their ongoing rhythms due to interaction, serving as a reliable marker of information flow between the elements of a system (Rosenblum *et al.* 2001; Burgess 2013). Thus, and this is key, inter-brain phase synchronization of neural activity potentially indicates functional integration across brains.

Nevertheless, ‘false’ synchronization between brain signals might appear, such as when oscillators are driven by an external influence, or when there is a coincidental phase relationship between individual rhythms. Therefore, careful consideration must be taken before formulating conclusions about their explanatory power (Burgess 2013). Induced synchrony (as opposed to true synchronization) could occur when participants are exposed to the same perceptual stimulus or exhibit similarity of movements, even without actually interacting with one another (Kaneshiro *et al.* 2020). Therefore, it can be difficult to conclude if synchrony is due to similar dynamics of individual brains driven by a shared perceptual context or if it is an emergent property of social interaction.

Fortunately, most hyperscanning studies today do not simply measure phase synchronization between individuals but compare it between different experimental conditions. To control for induced synchrony, experimental conditions remain identical in every way possible, except for one where participants are socially engaged and another when they are not (Burgess 2013). Many studies additionally use random pair analysis to account for spurious synchronization due to a shared environment (Osaka *et al.* 2015; Toppi *et al.* 2016). This analysis compares brain signals from real pairs of subjects (from those who did interact during the task) with signals from ‘random’ or ‘non-pairs’ (from pairs generated randomly based on their role in the task and not the actual pair they were a part of). By doing so, only the emergent neural synchronization that was due real interaction survives the analysis (Bilek *et al.* 2015; Osaka *et al.* 2015; Toppi *et al.* 2016).

## True synchronization is associated with cooperation

Besides revealing that synchronized brain activity is neither due to a shared environment (Dikker *et al.* 2017) nor to similarities in stimulus input or motor output (Sänger *et al.* 2012; Pérez *et al.* 2017), EEG-based hyperscanning studies show that functional links appear across participant’s brains during cooperation, but not during competition or individual—yet simultaneous—task performance (De Vico Fallani *et al.* 2010; Mu *et al.* 2016; Sinha *et al.* 2016; Mu *et al.* 2017; Szymanski *et al.* 2017; Balconi and Vanutelli 2018a, b) (note that this link is not only observed with EEG-based hyperscanning; see Box 1). For example, pilots and co-pilots in flight simulations (where the environment remains the same, but the need for cooperation varies throughout the task) exhibit high inter-brain connectivity during cooperative phases (takeoff and landing); such interconnections break down during cruise, when the two participants act independently (Toppi *et al.* 2016). Another clue that such findings are not due to confounding factors (Burgess 2013) but are instead a marker of real synchronization, connectivity between random couples proved non-significant compared to real couples (Toppi *et al.* 2016). More interestingly, non-cooperative interactions can be predicted during the decision-making phase of a computerized cooperation–competition game, where individuals can either decide to cooperate, defect `or choose a ‘tit-for-tat’ strategy (punish the other player for previous non-cooperative behavior). Prior to making the decision, defector couples already show significantly less inter-brain connectivity than couples playing cooperative or tit-for-tat strategies (De Vico Fallani *et al.* 2010). Interestingly, it has been suggested that this task requires a higher understanding of the other’s intentions when participants decide to cooperate or punish (Czeszumski *et al.* 2020).

**Box 1. Beyond EEG**

The type of results described in this article is not limited to the EEG-based hyperscanning technique; MEG, fMRI and fNIRS hyperscanning studies reveal similar findings. Reviewing them all is beyond the purpose of this work. However, we recognize their importance in providing a more complete picture of the brain’s activity during social interaction and emphasize the relevance of future multimodal recording and analysis.

Of particular relevance for oscillatory dynamics, fNIRS-based hyperscanning studies, which do not directly register electrical dynamics but can uncover locally phase-locked neural behaviour, show that higher inter-brain neural synchronization is observed when, for example, participants play a computer game side by side and are required to cooperate, but not when they are required to play against each other (Cui *et al.* 2012).

The pioneering study by Funane *et al.* (2011) revealed that the spatiotemporal coherence of inter-brain signals in paired participants was associated with cooperative performance. Participants were instructed to mentally count to 10 after an auditory cue, and then simultaneously press a button. When the brain-activity patterns during the counting period were more synchronized, the time interval between their button-presses was shorter, a result not explained in terms of a motion artefact.

Greater neural synchronization also appeared between subjects completing a puzzle together, compared to when the same subjects completed identical puzzles individually, or watched others complete the puzzle (in front of them or through video recording) (Fishburn *et al.* 2018). A similar study revealed inter-brain synchronization between two individuals when singing together, but not when singing individually yet close to each other (this effect was not observed in random pairs) (Osaka *et al.* 2015).

Additionally, not only does cooperation foster inter-brain synchronization; it appears that believing you are ‘part of the same team’ has also an effect on hyper-connectivity. In a study with four participants playing a card game in two competing teams (where, to control for motor activity, experimenters assisted the participants on moving the cards), strong functional connectivity was observed between subjects belonging to the same team, but not between subjects from different teams (Astolfi *et al.* 2010). Similarly, pairs exposed to a context that represents an in-group threat have higher levels of inter-brain synchronization during a coordination task than those exposed to out-group threats or in-group no-threat control conditions. Importantly, this connectivity only appears when pairs are required to coordinate with a human partner and not a computer (Mu *et al.* 2017).

Moreover, a recent study revealed that participants playing a cooperation game face-to-face exhibit differences in brain-to-brain synchronization when they believe they are interacting with each other compared to when they believe their interaction is with a computer. In this experimental setup, the prompts ‘your partner is a human’ and ‘your partner is a computer’ were provided before each condition (human–human or human–machine), and every dyad went through both conditions in the same session. Even though in both conditions the interaction was with the partner, believing otherwise had a strong effect on hyper-connectivity (Hu *et al.* 2018). This may reflect the effects of different levels of engagement (Schilbach *et al.* 2013).

If, under correct experimental setups, hyperscanning studies are revealing reliable markers of oscillatory synchronization, then it appears that, just as in an individual brain, the dynamics of neural activity during interaction could provide functional integration between the interacting parts—across brains (Kelso and Engstrom 2006; Stevens and Galloway 2016, 2017; Stevens *et al.* 2017). This is supported by one of the most interesting, yet lesser explored aspects of EEG-based hyperscanning studies: an association between inter-brain neural synchronization and participants’ conscious experiences related to social cohesion.

## Inter-brain synchronization and subjective experience of social closeness

Although several experimental paradigms do not explicitly take into account participant’s experience (Balconi and Vanutelli 2017), those that do have yielded interesting results. First, feelings of cooperativeness between participants appear to mediate the level of neural synchronization during cooperation (Hu *et al.* 2018). Additionally, feelings of engagement, affinity, empathy and social closeness can be predicted by the level of inter-brain synchronization (Dikker *et al.* 2017; Bevilacqua *et al.* 2019). Neural synchronization across pairs is also negatively associated with an individual’s reported attachment anxiety (Kinreich *et al.* 2017) and experience of pain, while it is positively associated with accurately rating another person’s pain experience (Goldstein *et al.* 2018). Interestingly, the feeling of another person’s touch or pain has been repeatedly reported in intra-brain studies, where a person’s own affective pain circuitry is activated while viewing another person receive painful stimuli (Hari and Kujala 2009). More importantly, shared pain experience has also been related to self-other confusion (Derbyshire *et al.* 2013) and appears to be absent in people in the autistic spectrum disorder (Minio-Paluello *et al.* 2009), which further supports brain-to-brain synchronization as a mechanism for sharing a social world (Hasson *et al.* 2012).

Moreover, the nature of the association between participants has an effect on neural synchronization (Pan *et al.* 2017) and the context under which it appears. During naturalistic nonverbal social interaction (gaze and positive affect), inter-brain neural synchronization appears selectively among romantic couples and not among strangers (this result is unrelated to other factors, such as verbal communication) (Kinreich *et al.* 2017).

These findings, summarized in Table 1, are consistent with behavioural studies that reveal that movement synchronization plays an important role in social cohesion (McNeill 1997; Valdesolo *et al.* 2010; Valdesolo and DeSteno 2011) and is associated with higher levels of empathy (Goldstein *et al.* 2017), less sensitivity to pain perception (Cohen *et al.* 2010) and experiences of self-other merging (Rabinowitch and Knafo-Noam 2015; Novembre *et al.* 2016; Galbusera *et al.* 2019)—on being ‘on the same wavelength’ with someone (Hari and Kujala 2009). More importantly, the changes in an individual’s conscious experience of social closeness that accompany hyper-connectivity are relevant for understanding the interactive nature of human cognition within a ‘second-person neuroscience’ (Schilbach *et al.* 2013; Varela *et al.* 2017) and can have important implications for our understanding of the social dimension of human consciousness and for better treating its disorders (Schilbach 2016; Redcay and Schilbach 2019).

**Table 1.** List of studies mentioned includes EEG- and fNIRS-based hyperscanning methodologies

Authors (year)	Method	Paradigm	Task	N	Frequencies recorded	Frequencies synchronized	Regions	Analysis	Findings
Hu *et al.* (2018) ^a^	EEG	Cooperation	Prisoner’s Dilemma game, with high or low cooperation indexes and conditions of (believed) human-human or human-machine interaction.	30	Theta, alpha, beta, gamma	Theta, alpha	Theta: fronto-central, Alpha: centro-parietal.	PLVn	Higher cooperation rates and greater inter-brain synchrony were present when participants believed to be interacting with a human than with a machine. In human-human conditions, inter-brain coupling was higher in contexts with a high cooperation index. Participants’ reports of perceived cooperativeness mediated the relationship between game context (high or low cooperation) and alpha inter-brain synchrony.
Bezerianos *et al.* (2015)	EEG	Cooperation	Computer-based piloting task.	8	Delta, theta, alpha, beta, gamma	Not evaluated	Centro-parietal and frontal.	PDC	Cross-brain coupling increased as task difficulty increased.
Sinha *et al.* (2016)	EEG	Cooperation	Computer-based cooperation-competition game.	24	Theta, alpha, beta, gamma	Alpha, beta	Central and parietal.	PCC	Inter-brain synchrony between the subjects was significantly higher when they cooperated with each other compared to the competitive scenario.
Toppi *et al.* (2016)	EEG	Cooperation	Flight simulation (takeoff, cruise and landing).	12	Theta, alpha	Theta, alpha	Alpha and theta: Fronto-parietal and centro-parietal. Theta: parieto-parietal.	PDC	A denser pattern of interconnections linking the dyads’ brain activities appeared during the two cooperative flight phases (takeoff and landing) with respect to the non-cooperative cruise phase (cruise). There was a density modulation according to the degree of cooperation between the two pilots. Results sustained random pair analysis.
Astolfi *et al.* (2010)	EEG	Cooperation	Card Game (Scopa).	14	Theta, alpha, beta, gamma	Alpha, beta, gamma	Prefrontal cortex, anterior cingulate cortex and parietal cortex.	PDC	Strong estimated functional connectivity was found in subjects belonging to the same team but not in subjects belonging to different teams. The functional connectivity links found were directional (i.e. the signals from the second players revealed a statistically significant Granger-causality with signals of the first players of the same team).
De Vico Fallani *et al.* (2010)	EEG	Cooperation	Iterated Prisoner’s Dilemma game (dyads with option to defect, cooperate or choose a tit-for-tat strategy in each trial).	58	Theta, alpha, beta, gamma	Theta, alpha, beta, gamma	Frontal and pre-frontal.	PDC	Non-cooperative interactions can be predicted during the decision-making phase. Prior to making the decision, defector couples already show significantly less inter-brain connectivity than couples playing cooperative or tit-for-tat strategies.
Balconi and Vanutelli (2018)	EEG	Competition	Computer-based competition task.	30	Delta, theta, alpha, beta	Delta, theta	Prefrontal.	PCC, ANOVA	Pair’s task performance (response times and error rates) was improved during competitive tasks with respect to control condition, with further improvement after receiving reinforcing feedback. Inter-brain functional connectivity was reduced during competition.
Mu *et al.* (2017)	EEG	Coordination	Mental counting task (with partner or with computer) after reading a text about in-group or out-group societal threats (with in-group no-threat control).	90	Delta, theta, alpha, beta, gamma	Gamma	Frontal, central and parietal.	PLVn	Greater inter-brain synchrony between participants under an in-group threat, which correlated with greater task coordination. Synchrony not present when each individual would try to mentally coordinate counting with a computer. Inter-brain synchrony mediated the effect of ingroup threat on interpersonal coordination.
Mu *et al.* (2016)	EEG	Coordination	Mental counting task.	68	Alpha	Alpha	Central and posterior.	PLVn	Dyads showed smaller interpersonal time lags of counting and greater inter-brain synchrony during the coordination task compared to control task. These effects were observed in female but not male dyads.
Mu *et al.* (2016)	EEG	Coordination	Mental counting task (with intranasal oxytocin vs placebo administration).	60	Alpha	Alpha	Central and posterior.	PLVn	Intranasal oxytocin (vs placebo) administration in male dyads improved interpersonal behavioral synchrony in both the coordination and control tasks but specifically enhanced inter-brain neural synchrony during the coordination task.
Szymanski *et al.* (2017)	EEG	Joint attention	Enumeration visual search task.	52	Delta, theta, alpha, beta	Delta	Frontal, central and parietal.	PLVn, IPC	Higher phase synchrony when doing the task together (joint attention) than when doing the same task individually (individual attention). Team efficiency could be predicted by measures of synchronization during dyadic performance.
Balconi and Vanutelli (2018).	EEG	Joint attention	Sustained selective attention task (cooperation of speed and accuracy was asked of each pair of subjects).	32	Delta, theta, alpha, beta	Delta, theta	Left prefrontal.	PCC, ANOVA	Cooperation correlated positively with inter-brain synchrony. External positive feedback increased both behavioural (higher reaction time and lower error rate) and brain synchronization.
Dikker *et al.* (2017) ^a^	EEG	Joint attention	High school students engaged in a semester during regular classroom activities.	12	Alpha	Alpha	Not reported.	TI	Brain-to-brain synchrony predicted classroom engagement and social dynamics (group affinity, empathy and social closeness). Joint attention, and not passive co-presence, predicted brain-to-brain synchrony.
Bevilacqua *et al.* (2019) ^a^	EEG	Joint attention	High school students in class under different teaching styles (videos and lectures).	12	Alpha	Alpha	Not reported.	TI	Brain-to-brain synchrony between teachers and students varied as a function of student engagement as well as teacher likeability. Students who reported greater social closeness to the teacher showed higher brain-to-brain synchrony with the teacher. This was only the case for lectures (compared to videos)—that is, when the teacher was an integral part of the content presentation.
Sänger *et al.* (2012)	EEG	Joint action	Playing guitar in duets in a leader-follower fashion.	24	Delta, theta	Delta, theta	Frontal and central.	PLVn, IPC	Phase locking and both within-brain and between-brain phase-coherence connection strengths were enhanced during periods that put particularly high demands on coordination. Phase locking was modulated in relation to the assigned musical roles of leader and follower.
Dumas *et al.* (2010)	EEG	Joint action	Spontaneous imitation of hand movements.	18	Theta, alpha, beta, gamma	Alpha-mu, beta, gamma	Alpha-mu: right parietal, Beta: central and parieto-occipital, Gamma: fronto-central and parietal.	PLVn	Inter-brain synchronization corresponded with interactional synchrony.
Pérez *et al.* (2017)	EEG	Communication	Speaking and listening.	30	Delta, theta, alpha, beta	Delta, theta, alpha, beta	Alpha: fronto-central. Beta: fronto-temporal.	PLVt	Increased inter-brain synchrony present between participants while speaking and listening (compared to surrogate data). The activity of the listener would become entrained to that of the speaker. These effects were not an epiphenomenon of auditory processing.
Kinreich *et al.* (2017) ^a^	EEG	Positive affect	Nonverbal social behaviour (gaze and affect) between co-habiting romantic couples or strangers.	104	Delta, theta, alpha, beta, gamma	Gamma	Temporo-parietal.	SPC	Inter-brain synchrony found in couples, but not in strangers. Among couples, neural synchrony was anchored in episodes of gaze and positive affect. Strangers’ synchrony did not show significant differences between moments of gaze-no gaze or positive affect-no affect, but a correlation was observed between the amount of time strangers spent in social gaze or positive affect and their gamma synchrony. In couples, synchrony was negatively correlated with attachment anxiety reports. For both couples and strangers, brain-to-brain synchrony was unrelated to episodes of speech-no-speech, speech duration, or general content of conversation.
Goldstein *et al.* (2018) ^a^	EEG	Pain perception	Dyads (with a target and observer) under pain-no-pain and touch-no-touch conditions.	44	Alpha, beta	Alpha-mu	Target: central. Observer: right hemisphere.	Ccorr	Handholding during pain administration increased brain-to-brain synchrony, which correlated negatively with the target's pain perception and positively with the observer’s empathetic accuracy.
Pan *et al.* (2017) ^a^	fNIRS	Cooperation	Computer-based cooperation game.	98	N/A	N/A	Right superior frontal cortex.	WTC	Cooperative behaviour was higher in lover dyads compared to friend and stranger dyads. Lover dyads demonstrated increased inter-brain coherence, which also covaried with task performance.
Cui *et al.* (2012)	fNIRS	Cooperation	Computer-based cooperation-competition game.	22	N/A	N/A	Superior frontal cortex.	WTC	Coherence between brain activation patterns increased significantly during cooperation, but not during competition. This increase accompanied an increase in cooperation performance in the cooperation game.
Funane *et al.* (2011)	fNIRS	Cooperation	Cooperative button-press task (with prior individual mental count after auditory cue).	12	N/A	N/A	Pre-frontal cortex.	Covariance	Spatiotemporal coherence of inter-brain signals of paired participants associated with cooperative performance. When the brain-activity patterns during the counting period were more synchronized, the interval between their button-press times was shorter, a result not explained in terms of a motion artefact.
Osaka *et al.* (2015)	fNIRS	Coordination	Singing/humming.	58	N/A	N/A	Left inferior frontal cortex.	WTC	Significant increase in neural synchronization for cooperative singing or humming (regardless of being face-to-face or face-to-wall) compared to singing or humming alone or listening to the other participant sing or hum.
Fishburn *et al.* (2018)	fNIRS	Joint attention	Completing a puzzle (together or individually).	57	N/A	N/A	Prefrontal cortex.	PCC, WTC	Interpersonal neural synchronization was greater when completing the puzzle together than when completing the same puzzle individually, watching a pair complete the puzzle or watching a movie of other people completing the puzzle.

^a^ The studies that took into account the participants’ subjective experience or their relationship nature (e.g. lover dyads vs strangers).

PLVn, phase locking value (trial averaged); PLVt, phase locking value (time averaged); IPC, interbrain phase coherence; PCC, Pearson correlation coefficient; ANOVA, analysis of variance; PDC, partial directed coherence; TI, total interdependence; SPC, spearman correlation coefficient; CCorr, circular correlation; WTC, wavelet transform coherence; N/a, non-applicable.

## Beyond individualist theories of consciousness

If the basis of consciousness is at the level of large-scale interactions of neural oscillatory activity (Crick and Koch 1990; Thompson and Varela 2001; Varela *et al.* 2001; Ward 2003; Engel *et al.* 2016), the modifications of oscillations that appear during meaningful social interaction—and their relation to the experiences of social closeness—result in a shift in our understanding of human consciousness. Although a still debated proposal (Clark 2009, 2012; Ward 2012; Kirchhoff 2014; Kirchhoff and Kiverstein 2019), an ‘extension of consciousness’ could be possible in light of these findings.

Enactive and participatory approaches to social cognitive neuroscience view cognition as an interactive process, challenging the individualist notion of the human mind (Clark and Chalmers 1998; De Jaegher and Di Paolo 2007; Torrance 2009; Kirchhoff 2014; Varela *et al.* 2017; Froese 2018; Kirchhoff and Kiverstein 2019). These approaches establish the association between an individual, the world and others in terms of non-linear interactive dynamics (Beer 2000; Froese and Ikegami 2013). The boundaries that distinguish self from other, instead of being fixed and hard won, are under constant renegotiation (Clark and Chalmers 1998; Kirchhoff 2014; Kirchhoff and Kiverstein 2019); they are observed as a result of self-organizing processes, which can also occur at a social level depending on the nature of the interaction (De Jaegher and Di Paolo 2007; Galbusera *et al.* 2019). Interestingly, the inter-brain synchronization found—at least in the studies considered in Table 1—does not appear to have an overall frequency-, region- or task-specificity. This potentially places embodied interpersonal interaction in general as playing a fundamental role in shaping joint experience and moves social cognition away from the head (Thompson and Varela 2001; Froese and Ikegami 2013).

Considering this, the impact of meaningful social interaction on individual’s self-organizing processes (i.e. ongoing neural oscillations) and, more importantly, the appearance of true phase synchronization between brains (a key coordination variable between interacting dynamical systems Kelso and Engstrom 2006) support the suggestion that when several agents interact, a form of social self-organization could take place, with properties irreducible to the individuals (De Jaegher and Di Paolo 2007; Froese *et al.* 2014; Galbusera *et al.* 2019). Consequently, the boundaries consciousness could also be under renegotiation during meaningful social interaction.

The main objection to this argument, put forward by Clark (2009), is that consciousness cannot extend outside an individual brain since it requires processes occurring on such a fast temporal scale that neural activity proves to be the only adequate ‘vehicle’. Moreover, the person’s body will act as a ‘low-pass filter’, ultimately ‘screening off’ all non-neural body and environmental elements from being part of the material realizers of conscious experience. Therefore, entering signals can only play a casual, yet not constitutive, role (Clark 2009).

First, to be clear, when referring to an ‘extension of consciousness’, we are not suggesting that consciousness does not have any neural basis. Human consciousness requires mechanisms occurring within the brain (Thompson and Varela 2001; Engel *et al.* 2016); nevertheless, neural activity is also embodied in an individual’s interaction in and with the world (Buzsáki 2006; Noë 2009), including with other people (Hari and Kujala 2009). More importantly, inter-brain neural synchronization, as revealed through EEG-based hyperscanning studies, appears in all oscillatory frequency bands, including the faster gamma frequency (Astolfi *et al.* 2010; Dumas *et al.* 2010; Kinreich *et al.* 2017; Mu *et al.* 2017). There is therefore no empirical reason to limit the basis of conscious experience to very fast neural activity occurring within a single brain.

Additionally, communication within a single brain uses the hierarchical nesting of oscillations, where faster frequencies are embedded in, and modulated by, slower rhythms (Buzsáki 2006; Buzsáki and Draguhn 2004; Bonnefond *et al.* 2017). This line of reasoning can be extended to the even slower timescales of embodied action in the world (Haken 1980; Kelso 2009; Van Orden *et al.* 2012; Haken 2013). In this sense, even if the body acted as a ‘low-pass filter’, it remains possible for embodied social interaction to work as a slower rhythm in which neuronal oscillations become nested across two or more people (Hasson *et al.* 2012), and thus foster ‘interpersonal synergies’ (Riley *et al.* 2011; Hasson *et al.* 2012).

By eliminating Clark’s frequency-based objection, we propose that the boundaries of the conscious mind could also be subject to constant renegotiation during an individual’s interaction with his/her environment and with others, pointing to a mechanism that neurally binds us together and opens us up to an extended conscious mind in social interaction (Kelso and Engstrom 2006). An upshot of this proposal is that it can potentially validate our most intimate experiences: when we become aware that ‘we’ are sharing a moment with someone else, it is no longer necessarily the case that we are fundamentally separated by our distinct heads—we could really be two distinct individuals sharing in one and the same unfolding experience (Froese 2018).

The consequence of Clark’s claim that fast temporal integration of neural activity delimits the basis of consciousness, when viewed from the latest hyperscanning evidence, is that this basis can indeed become extended across two brains during social interaction. For Clark, and for us, this is a constitutive claim, not a causal claim. Importantly, this does not necessarily mean that individual perspectives will be obliterated and merged into a single supra-individual perspective (Stapleton and Froese 2015). We propose that the fact that the basis of an individual’s perspective now integrates some neural activity from another person’s brain provides a suitable explanation for the qualitative experiential transition from a purely first-person perspective to a second-person perspective, which is also sometimes referred to as the ‘we’ perspective (Zahavi 2015). If this proposal is on the right track, then there could be a genuinely collective basis to collective intentionality (Searle *et al.* 1990), thereby overcoming difficulties stemming from the individualist assumption of traditional theories in philosophy. There are also experimental consequences: presumably, the larger the cross-brain neural integration, the larger the qualitative sense of sharing an experience together, and this could potentially be tested.

## Testing the hypothesis: a possible experimental setup

We have proposed that inter-brain phase synchronization of neural oscillatory is a candidate mechanism for the conscious extended mind, potentially explaining the known association of synchronization with the feeling of being together with someone else. Although consciousness is notoriously difficult to investigate scientifically, here we suggest that our proposal could be experimentally tested through transcranial alternating current stimulation (tACS). Arguably, the best method known to date that assesses the causality of brain oscillations in human cognition (Cohen 2014), tACS can directly modulate the ongoing rhythms of neural activity in an individual brain by passing an electrical current between two electrodes placed on the scalp, entraining specific oscillations and thereby influencing cognitive processes ( Cohen 2014; Zaehle *et al.* 2010; Helfrich *et al.* 2014; Herrmann *et al.* 2013 ) and large-scale network dynamics (Ali *et al.* 2013).

When used in individual brains, this method has been reported to induce changes in subjective experience (Meiron and Lavidor 2014), such as self-awareness (Voss *et al.* 2014). In addition, its therapeutic applications on individuals with disorders of consciousness, such as on patients with schizophrenia, are already demonstrated (Kallel *et al.* 2016; Mellin *et al.* 2018; Ahn *et al.* 2019). These findings support this method’s relevance for investigating the extension of consciousness during social interaction.

From the available literature it appears that simultaneous brain stimulation, or hyper-tACS, is associated with an enhancement of interpersonal movement synchrony (Novembre *et al.* 2017). In a study conducted by Novembre *et al.* (2017), in-phase 20 Hz stimulation across two individuals’ motor cortices enhanced behavioural synchrony in a joint finger-tapping task, compared with anti-phase or sham stimulation. This was seen even after controlling for stimulus induced synchronization and performing random pair analysis (Novembre *et al.* 2017).

Under a setup of social interaction similar to those utilized in the aforementioned EEG-based hyperscanning studies (where true synchronization between participant’s brains takes place (Burgess 2013), hyper-tACS could be used to either enhance or diminish pre-existing inter-brain synchronization during a cooperative task to study its effects on the participant’s subjective experience of social connectedness and engagement. We expect that hyper-tACS conditions that foster brain-to-brain synchronization during cooperative action would lead to higher experienced cohesion, compared to hyper-tACS conditions that inhibit neural synchronization across participant’s brains.

tACS-EEG co-registration is still in its early stages, but promises to ‘open a new frontier in oscillatory brain rhythms investigations’ (Feurra *et al.* 2012). Even though there is no established hyper-tACS/EEG method to date, it has proven an effective method for manipulating on-going inter-brain phase synchronization during joint action (Novembre *et al.* 2017; Szymanski *et al.* 2017). However, results are still inconclusive. As opposed to Novembre’s study, Szymanski *et al.* (2017) found that only dyadic performance asynchrony, compared to dyadic synchrony and individual synchronization to a metronome, was modulated by hyper-tACS. Due to tACS dependence on the power of individual endogenous oscillations at the targeted frequency (Ruhnau *et al.* 2016), this study’s electrode placement left room for individual differences within each dyad, so unique dyad differences might have resulted in an unprecise inter-brain phase synchronization.

Further hyper-tACS studies, with more precise stimulation protocols, are needed in order to ensure that oscillations in the same frequencies are synchronized in the brains of individuals engaged in social interaction (Szymanski *et al.* 2017). Only then could the subjective experience of social connectedness be manipulated in a valid experimental setup. If this turned out to be the case, it would establish inter-brain neural synchronization as the basis for the conscious extended mind, at least for situations where it matters most—during meaningful social interaction.

## Conclusion

An influential position in the philosophy of mind articulated by Clark had appealed to the lack of empirical evidence regarding very fast binding processes outside the nervous system to conclude that the possibility of the conscious extended mind is at best unproven. As we have shown, the current evidence no longer supports such a sceptical assessment. With these theoretical doubts removed, we conclude that the time is right to marshal the best contemporary hyperscanning practices in cognitive neuroscience in order to move beyond the still widespread traditional assumption that all aspects of consciousness are necessarily private and first-person singular. It is time to bring the sciences of the mind in line with the social nature of human experience. 

*Conflict of interest statement*. None declared.
